# HAEMOPTYSIS - A RARE PRESENTATION OF AORTIC ANE URYSM

**DOI:** 10.4103/0970-2113.44133

**Published:** 2008

**Authors:** Girija Nair, Savita Jindal, Abehishek Chandra, Shivani Swami, Pankaj Garg

**Affiliations:** Padamshree Dr. D. Y.Patil Hospital & Research Center, Nerul, Navi Mumbai

**Keywords:** Aortic aneurysm, Haemoptysis, Mediastinal mass

## Abstract

We report a case of a 65 year old man who presented with haemoptysis. X-ray chest PA view shows mediastinal widening suggestive of mediastinal mass. A CT scan of thorax shows a large aneurysm of ascending aorta & arch of aorta. This an-eurysm is eroding the trachea and abutting the sternum and vertebrae.

The patient is VDRL reactive. Haemoptysis is a rare presentation of aortic aneurysm.

## INTRODUCTION

Haemoptysis in aortic aneurysm is very rare, caused by erosion of trachea by the aneurysm or rupture of aneurysm into the lung.

A patient who presented with haemoptysis had a large mediastinal mass on X - Ray chest.CT Scan demonstrated a large aneurysm of ascending aorta and arch of aorta.

The patient is VDRL Reactive.

## CASE REPORT

A 65 year old farmer, smoker complains of blood in sputum daily around 5-6 cc in amount for 2 months. The patient does not have any other complaint.

On Examination -

Blood Pressure - 168/100 mm of Hg

Tracheal tug is present No clubbing, no cervical or axillary lymphadenopathy, Arm span is less than height.

Upper respiratory tract is normal.

No signs of Superior vena cava obstruction.

On auscultation

Respiratory System - Bilateral Breath sounds are equal No adventitious sound present

Cardiovascular System - Heart sounds normal No murmur of Aortic Regurgitation

**Fig 1:**
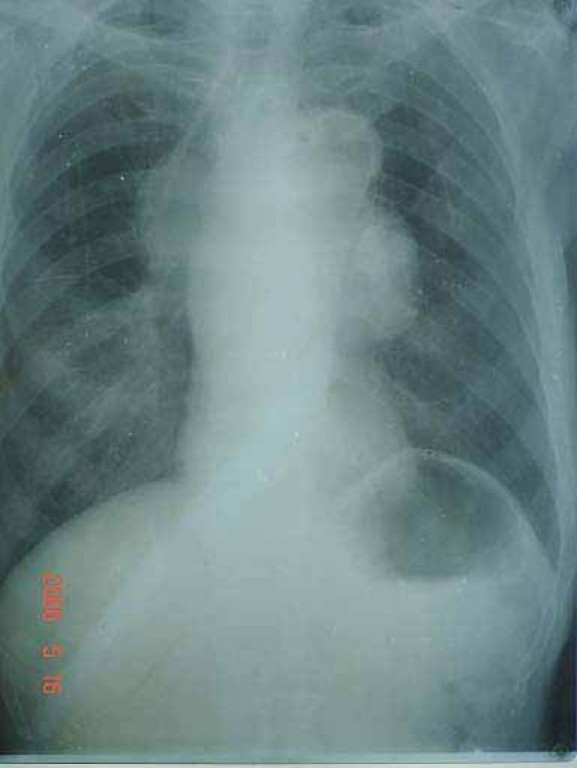
Chest X-Ray PA view showing mediastinal mass

Chest Xray posteroanterior view shows mediastinal widening which is suggestive of mediastinal mass (Fig 1). CT Thorax is suggestive of mass lesion measuring approxmately 13.3cm* 8.4cm *9.5 cm in anterior and middle mediastinum. It is Contiguous with walls of ascending thoracic aorta & arch of aorta which are grossly dilated and show mural calcification.

**Fig 2:**
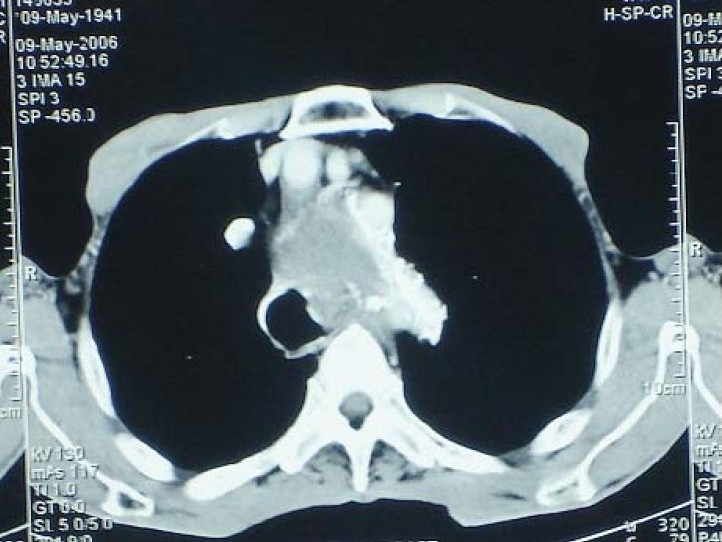
CT Thorax showing the aneurysm bulging into the left wall of Trachea.

**Fig 3:**
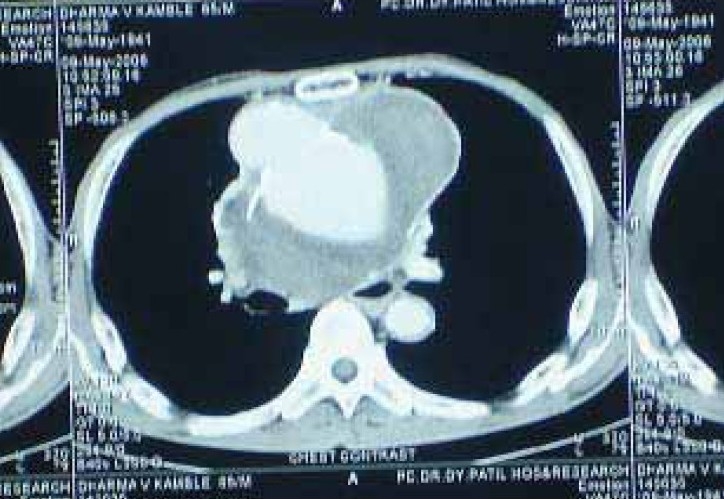
CT Thorax showing aneurysm abutting sternum and vertebrae, calcification of aortic wall & thrombus in aorta

There is large thrombus in it. There is focal bulge with narrowing of tracheal lumen just proximal to carina (Fig 2) The lesion is compressing the trachea, carina, the right and left mainstem bronchi and oesophagus displacing them to the right. The lesion is also abutting the anterior margin of the dorsal vertebrae and the right postero lateral sternum at D3-D7 levels (Fig 3). The lesion is abutting and compressing the azygous vein, superior vene cava, the superior pulmonary veins, the origins of the right and left pulmonary arteries and the main pulmonary artery, no thrombosis of these vessels VDRL is Reactive. The complete blood count, liver function test, renal function test are within normal limits.

The fasting blood sugar is 122mg/dl and post prandial blood sugar is 218mg/dl. Lipid profile is within normal limits.

## DISCUSSION

Review of literature comes up with very few cases of aortic aneurysm presenting with haemoptysis. It can occur due to tracheal erosion by constant pulsation of aneurysm of aorta. A case of aneurysm of descending aorta which ruptured into left upper lobe leading to massive haemoptysis is described in literature.^1^ Atheromatous abdominal aortic aneurysm can rupture into a lobe of the lung leading to haemoptysis.^2^

Other symptoms of aneurysm may be dysphagia, stridor, hoarseness of voice due to compression of aneurysm on oesophagus, trachea, and recurrent laryngeal nerve respectively. Patients with large aneurysms like the one we are reporting may also complain of chest pain due to bony erosion of sternum and vertebrae. Causes of aneurysm are syphilis, atherosclerosis, cystic medial necrosis, Marfans syndrome, Ehler-Danlos syndrome^3^. Syphilitic periaortitis & mesoaortitis damage elastic fibres, resulting in thickening & weakening of aortic wall. Approximately 90% of syphilitic aneurysm are located in ascending aorta or arch of aorta.^3^

In our case, there is bulge in left wall of trachea just proximal to carina due to aneurysm. Cause of haemoptysis in our case may be due to erosion of trachea by large aneurysm. Patients with thoracic aortic aneurysm should receive beta blocker therapy. Operative repair with placement of a prosthetic graft is indicated in patients with symptomatic thoracic aortic aneurysm & in those in whom the aortic diameter is> 6 cm.^4^
